# Comparing Patient’s Confidence in Clinical Capabilities in Urology: Large Language Models Versus Urologists

**DOI:** 10.1016/j.euros.2024.10.009

**Published:** 2024-10-23

**Authors:** Nicolas Carl, Lisa Nguyen, Sarah Haggenmüller, Martin Joachim Hetz, Jana Theres Winterstein, Friedrich Otto Hartung, Britta Gruene, Jakob Nikolas Kather, Tim Holland-Letz, Maurice Stephan Michel, Frederik Wessels, Titus Josef Brinker

**Affiliations:** a Digital Biomarkers for Oncology Group, German Cancer Research Center (DKFZ), Heidelberg, Germany; b Department of Urology, University Medical Center Mannheim, Ruprecht-Karls University of Heidelberg, Mannheim, Germany; c Medical Faculty Mannheim, Ruprecht-Karls University of Heidelberg, Mannheim, Germany; d Medical Faculty Heidelberg, Ruprecht-Karls University of Heidelberg, Heidelberg, Germany; e Medical Faculty Carl Gustav Carus, Else Kroener Fresenius Center for Digital Health, TUD Dresden University of Technology, Dresden, Germany; f Department of Biostatistics, German Cancer Research Center (DKFZ), Heidelberg, Germany

**Keywords:** Clinical trial, Generative artificial intelligence, Implementation science, Large language models, Patient interaction

## Abstract

**Background and objective:**

Data on interaction of patients with artificial intelligence (AI) are limited, primarily derived from small-scale studies, cross-sectional surveys, and qualitative reviews. Most patients have not yet encountered AI in their clinical experience. This study explored patients’ confidence in AI, specifically large language models, after a direct interaction with a chatbot in a clinical setting. Through hands-on experience, the study sought to reduce potential biases due to an anticipated lack of AI experience in a real-world urological patient sample.

**Methods:**

A total of 300 patients scheduled for counseling were enrolled from February to July 2024. Participants voluntarily conversed about their medical questions with a GPT-4 powered chatbot, followed by a survey assessing their confidence in clinical capabilities of AI compared with their counseling urologists. Clinical capabilities included history taking, diagnostics, treatment recommendation, anxiety reduction, and time allocation.

**Key findings and limitations:**

Of the 292 patients who completed the study, AI was significantly preferred to physicians for consultation time allocation (*p* < 0.001). However, urologists were overwhelmingly favored for all other capabilities, especially treatment recommendations and anxiety reduction. Notably, age did not influence patients’ confidence in AI. Limitations include a potential social desirability bias.

**Conclusions and clinical implications:**

Our study demonstrates that urological patients prefer AI as a powerful complement to—rather than a replacement for—human expertise in clinical care. Patients appreciated the additional consultation time provided by AI. Interestingly, age was not associated with confidence in AI, suggesting that large language models are user-friendly tools for patients of all age groups.

**Patient summary:**

In this report, we explored how patients feel about using an artificial intelligence (AI)-powered chatbot in a medical setting. Patients interacted with the AI for medical questions and compared its skills with those of doctors through a survey. They appreciated the AI for providing more time during consultations but preferred doctors for other tasks, for example, diagnostics, recommendation of treatments, and reduction of anxieties.

## Take Home Message

While patients value artificial intelligence (AI) for extended consultation time, they trust human physicians more for crucial medical tasks. Confidence in AI showed no age differences, indicating that large language models are user-friendly tools for patients of all age groups.

## 1 Introduction

Large language models (LLMs) are very powerful human language processing machines with vast potential across a wide range of domains and applications [1], [2]. LLMs are artificial neural networks with billions of parameters trained on a vast spectrum of texts sourced from the Internet, which includes medical sources [3], [4]. Off-the-shelf LLMs excel in “zero-shot” tasks [5] such as extracting and structuring data, summarizing medical notes, improving the readability of reports, recommending treatments, and answering medical questions [3], [6], [7]. The application of LLMs for medical question answering (medQA) is culminating in the evaluation of medical board examinations, where LLMs are able to outperform humans [8], [9], [10], [11], [12], [13], [14]. By offering a dynamic and more direct approach to information retrieval, LLMs allow users to obtain specific answers without the need to read through entire articles, which could transform and improve how medical knowledge is assessed in health care by reducing barriers. It is assumed that LLMs could soon supplement or even replace search engines as primary sources of medical information for patients [15], [16], [17].

While LLMs show promise, the extent to which patients trust AI in their health care journey is less clear. To better understand this, several studies have begun investigating patient confidence in AI within clinical workflows. Two cross-sectional surveys on patients’ confidence in AI within clinical workflows reveal a general preference for physicians over AI, but also indicate trust in AI-assisted care when supervised by a physician [18], [19]. These studies offer valuable insights but might be biased due to limited prior experience with AI technologies of the surveyed participants [18], [19]. Moreover, data on patient interactions with LLMs are sparse, with most evidence stemming from cross-sectional surveys [18], [19], [20], one interventional study with very limited sample size (*n* = 9) [21], and qualitative research evaluating online user reviews posted on AppStores [22]. In summary, LLMs are powerful tools that have proven high efficacy in silico across various domains and applications. However, there are limited data on in vivo evaluations of true benefits of AI, opinions, and patient-reported outcomes [23].

To address the expected lack of AI experience in a real-world urological patient sample, we designed a prospective clinical trial that introduced patients to LLMs in a controlled setting. The goal was to reduce biases by providing hands-on experience and to enable a realistic assessment of patient interactions with LLMs. Following this interaction, we conducted a survey to reassess patient confidence in AI compared with physicians.

## 2 Patients and methods

### 2.1 Reporting standards and ethics statement

To address the unique challenges of LLM research in health care applications, the TRIPOD-LLM [24] statement was followed as a reporting guideline, with a corresponding checklist provided in the Supplementary material. The study was approved by the institutional ethics review board of the Medical Faculty Mannheim, Ruprecht-Karls University of Heidelberg (proposal number: 2023-687). The study was conducted in accordance with ICH guidelines and the principles of the Declaration of Helsinki, with written informed consent obtained from all study participants. The study was officially registered with the German Clinical Trial Registry (DRKS-ID: 00034906).

### 2.2 Patient enrollment

Patients attending urological counseling for elective urological surgery at the University Medical Center Mannheim were prospectively enrolled onsite. The eligibility criteria included being 18 yr or older, excluding patients with cognitive impairments or psychiatric conditions. Informed consent was obtained from all participants.

### 2.3 Study design and procedure

**Fig. 1:**
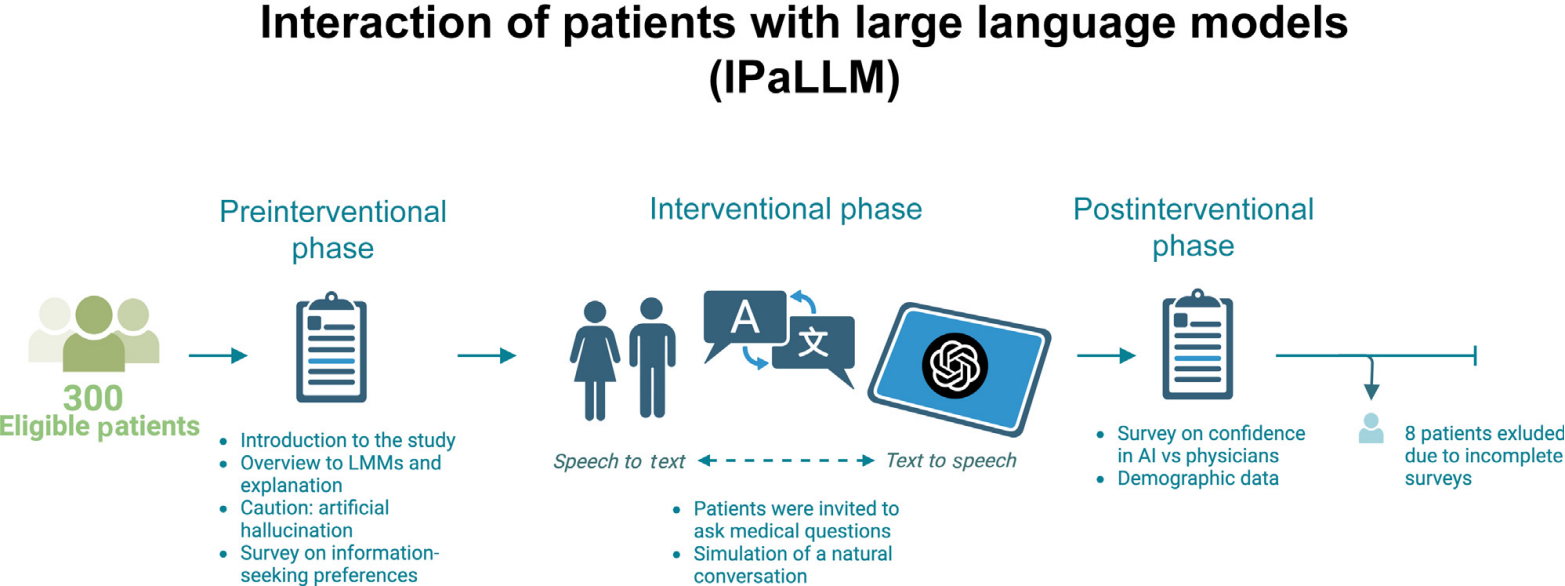
Study design and procedure. Participants received a guided introduction to LLMs and completed a preinterventional survey assessing their information-seeking preferences and AI affinity. In the interventional phase, patients then interacted with a GPT-4 powered chatbot in a controlled setting, asking medical questions relevant to their current clinical situation. In the postinterventional phase, participants filled out a survey evaluating their confidence in AI versus physicians. AI = artificial intelligence; LLM = large language model.

The study consisted of three phases: a preinterventional phase, an interventional phase with a guided chatbot conversation, and a postinterventional survey to assess patients’ perspectives on AI (see Fig. 1). We assumed little to no prior experience with AI technologies in elderly patients, a key demographic in urology. The study was designed to reduce barriers by providing a guided introduction to LLMs in a controlled setting. This approach aimed to build necessary media competence among participants.

#### 2.3.1 Preinterventional phase

Participants were provided with a description of the experiment, an overview of LLMs with an explanation of the general “prompt-answer” principle. Participants were advised that LLMs might produce “artificial hallucinations”—information that appears accurate but is actually incorrect. Following this, participants then completed a preinterventional survey to assess their medical information-seeking preferences, baseline knowledge, and affinity for AI.

#### 2.3.2 Interventional phase

Participants interacted with a GPT-4 powered chatbot (OpenAI) via a browser application on a tablet, using speech to text for input and text to speech for responses, simulating a natural conversation. Participants were encouraged to ask medical questions related to their current clinical concern. The chatbot was prompted earlier with keywords specific to the clinical concern (eg, prostate biopsy or nephrolithiasis). Participants were instructed to ask questions naturally, as they would with a physician or online. The experiment was conducted under controlled conditions, with study personnel available to correct any incorrect information provided by the chatbot.

#### 2.3.3 Postinterventional phase

Next supervisors left the room to ensure anonymity of the survey results. Participants then completed the postinterventional survey and a demographic questionnaire on the tablet. The survey, based on the one developed by Lennartz et al [19], was conducted in German. An English translation was provided for reporting purposes (see the Supplementary material). The entire process took approximately 30 min per participant. Afterward, any remaining questions or instances of artificial hallucinations were addressed by the study personnel to clarify potential misinformation.

### 2.4 Questionnaire development

We initially conducted a literature review on existing research, followed by six semistructured expert interviews with patients (*n* = 3) and board-certified urologists (F.W., F.O.H., and B.G.) to gather detailed insights. Subsequently, the survey questionnaire and intervention were drafted and reviewed by the interviewed urologists and an AI expert (S.H.). Finally, the survey and chatbot intervention was tested by individuals with no professional background in AI (*n* = 20), to ensure clarity and consistency.

### 2.5 Data collection, analysis, and statistics

All data were collected via a tablet. The results were assessed on a numerical Likert scale ranging from 0 to 5. For analysis, it was assumed that the intervals between the Likert levels were identical. Despite the constrained nature of this variable, the central limit theorem allows us to consider mean values to be approximately normally distributed. We report the mean difference, *p* value, and Cohen's d for each capability to assess the effect size. Paired sample two-tailed *t* tests were used to compare mean confidence levels in physicians versus AI across various clinical capabilities, including history taking, diagnostics, treatment recommendation, guideline concordance, anxiety reduction, relevant information provision, and time allocation. As this is an exploratory trial, no adjustment for multiple testing was done.

To identify the predictors of confidence in AI, a multiple linear regression analysis was conducted, adjusting for variables such as age, use of LLMs for medQA, use of Google, confidence in online information sources, and affinity for AI. The β coefficients for estimated slopes, 95% confidence intervals (CIs; using the modified Wald method [25]), and *p* values were reported for each predictor. The overall significance of the model was determined by the model *p* value, with a significance level (alpha) set at 5% for all hypothesis testing. Data analysis was performed using RStudio (version 1.0.136; R Foundation for Statistical Computing, Vienna, Austria).

## 3 Results

### 3.1 Study sample represents a real-life distributed urological cohort

Out of 300 prospectively enrolled patients, 292 completed the study, yielding a response rate of 97.3%. The study cohort included 212 male and 71 female participants, with a median age of 64 yr (ranging from 18 to 96 yr). Detailed demographic characteristics are provided in Table 1. Patients were consulted for a broad range of urological areas, including uro-oncological surgery (eg, prostate cancer, kidney cancer, testicular cancer, and bladder cancer surgery), endourological surgery (eg, benign prostatic hyperplasia and urolithiasis), as well as reconstructive and transgender surgery. For a detailed description, please refer to the Supplementary material.

**Table 1:** Demographic results and information-seeking preferences^a^

Characteristic	Overall (*N* = 292)	Young adult (*n* = 23)	Adult (*n* = 28)	Senior (*n* = 96)	Elderly (*n* = 145)
Age (yr)	60.58 (15.7)	23.61 (2.9)	38.14 (5.6)	58.73 (4.0)	71.99 (5.4)
Sex					
Male	212 (73)	11 (48)	16 (57)	71 (74)	114 (79)
Female	71 (24)	12 (52)	11 (39)	24 (25)	24 (17)
Education					
1 (ISCED 0–1)	3 (1)	0 (0)	2 (7)	0 (0)	1 (1)
2 (ISCED 2–3)	83 (28)	8 (35)	9 (32)	30 (31)	36 (25)
3 (ISCED 4–6)	43 (15)	8 (35)	5 (18)	13 (14)	17 (12)
4 (ISCED 7–8)	84 (29)	2 (9)	8 (29)	26 (27)	48 (33)
Comorbidities					
Present	151 (52)	1 (4)	10 (35)	52 (54)	88 (60)
Living arrangement					
Alone	44 (15)	3 (13)	10 (36)	15 (16)	16 (11)
In cohabitation	187 (64)	14 (61)	13 (46)	63 (84)	97 (67)
Use of Google for medical information					
Yes	239 (82)	22 (96)	27 (96)	81 (84)	109 (75)
No	53 (18)	1 (4)	1 (4)	15 (16)	36 (25)
Frequency of Internet use for medical information					
Monthly	53 (18)	2 (9)	1 (3.6)	16 (17)	34 (23)
Weekly	100 (34)	9 (39)	13 (46)	34 (35)	44 (30)
Daily	139 (48)	12 (52)	14 (50)	46 (48)	67 (46)
Confidence in online sources					
Very low	38 (13)	6 (26)	1 (4)	16 (17)	15 (10)
Rather low	65 (22)	3 (13)	10 (36)	21 (22)	31 (21)
Neutral	142 (49)	11 (48)	12 (43)	49 (51)	70 (48)
Rather high	39 (13)	3 (13)	5 (18)	9 (9)	22 (15)
Very high	8 (3)	0 (0)	0 (0)	1 (1)	7 (5)
Affinity for artificial intelligence					
Very low	58 (20)	0 (0)	2 (7)	17 (18)	39 (27)
Rather low	95 (33)	6 (26)	7 (25)	36 (38)	46 (32)
Neutral	83 (28)	5 (22)	7 (25)	28 (29)	43 (30)
Rather high	46 (16)	8 (35)	11 (39)	14 (15)	13 (9)
Very high	10 (3)	4 (17)	1 (4)	1 (1)	4 (3)
Have you ever heard of ChatGPT or other LLMs?					
Yes	201 (69)	22 (96)	24 (86)	62 (65)	93 (64)
No	91 (31)	1 (4)	4 (14)	34 (35)	52 (36)
Have you ever used ChatGPT or other LLMs for medQA?					
Yes	22 (8)	6 (26)	6 (21)	6 (6)	4 (2)
No	259 (89)	17 (74)	21 (75)	87 (90)	134 (92)

AI = artificial intelligence; LLM = large language model; medQA = medical question answering; SD = standard deviation.

Demographics were separated by age groups including young adults (18–30 yr), adults (30–50 yr), seniors (50–65 yr), and elderly (>65 yr); age in years; educational levels according to the 2001 International Standard Classification of Education (ISCED); survey results (*n* = 292) on information-seeking preferences; AI affinity; and use of LLMs for medical questions.

For better readability, percentages in this table are rounded to whole numbers.

a Data are presented as *n*/*N* (%) or mean (SD).

### 3.2 Urological patients rely on Google for information and report limited experience with AI

To investigate information-seeking preferences among patients, we first assessed the preferred source of medical information, confidence in online sources, and affinity for AI technologies. This section presents the findings from the survey conducted in the preinterventional phase.

The majority of patients (239/292; 82%; 95% CI: 77–86%) used Google as the primary source of medical information, with nearly half of them (139/292; 48%; 95% CI: 41–53%) using it daily. More participants (103/292; 35%; 95% CI: 30–41%) responded to have “rather low” or “very low” confidence in online sources for medical information compared with “rather high” or “very high” confidence (47/292; 15.7%; 95% CI: 12–21%). Approximately half of the sample (153/292; 53%; 95% CI: 47–58%) indicated a rather low or very low affinity for AI technologies, while fewer participants (56/292; 19%) reported a rather high or very high affinity. Notably, 89% (259/292; 95% CI: 85–92%) of participants have never used LLMs for medQA. A significant difference in LLM usage for medQA was observed across age groups, with increased usage among “young adults” (six of 23; 26%) and “adults” (six of 27; 22%, *p* value for age group difference <0.001). Together, these data show that patients predominantly rely on Google in medical information seeking but exhibit low confidence in online sources. In addition, patients reported limited affinity for AI technologies, with minimal prior exposure to LLMs, especially senior and elderly patients, who comprise the majority of urological patients.

### 3.3 Patients have higher confidence in physicians than in AI for most clinical capabilities, except for allocated time

**Fig. 2:**
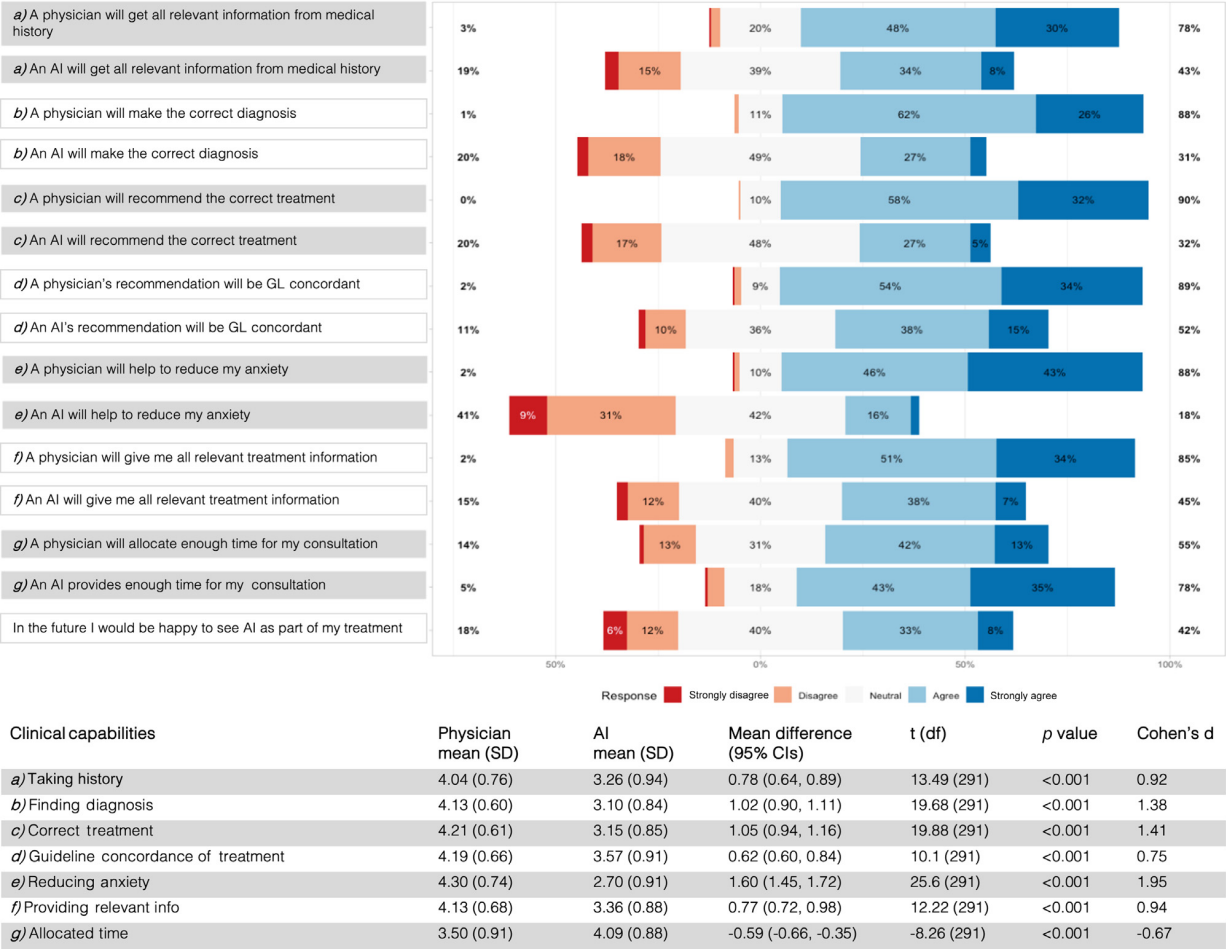
Overview of the postinterventional survey results. Patient confidence levels in physicians versus artificial intelligence regarding clinical workflow capabilities are displayed. The Likert plot displays the items and results of the t test to compare means. The survey was adapted from the study of Lennartz et al [19]. AI = artificial intelligence; CI = confidence interval; df = degrees of freedom; GL = guideline; SD = standard deviation.

Next, we investigated whether the confidence levels assigned by patients differ between AI and physicians. We compared mean confidence levels for clinical capabilities, including history taking, diagnostics, treatment recommendations, guideline adherence, anxiety reduction, provision of relevant information, and time allocation. The results from the postinterventional survey are displayed in Figure 2.

For medical history taking, physicians (mean = 4.04) were preferred to AI (mean = 3.26), with a mean difference of 0.78 (*p* < 0.001, Cohen's d = 0.92). In diagnostics, physicians (mean = 4.13) outscored AI (mean = 3.10) by 1.02 (*p* < 0.001, Cohen's d = 1.38). For treatment recommendations, physicians (mean = 4.21) were favored over AI (mean = 3.15), with a 1.05 difference (*p* < 0.001, Cohen's d = 1.41). For guideline concordance, the mean difference was 0.62 (*p* < 0.001, Cohen's d = 0.75), favoring physicians (mean = 4.19) over AI (mean = 3.57). Anxiety reduction showed the largest gap, with a difference between physicians (mean = 4.30) and AI (mean = 2.70) of 1.60 (*p* < 0.001, Cohen's d = 1.95). For providing relevant information, physicians (mean = 4.13) scored higher than AI (mean = 3.36), with a mean difference of 0.77 (*p* < 0.001, Cohen's d = 0.94). However, AI was preferred for allocated consultation time (mean = 4.09) over physicians (mean = 3.50), with a difference of –0.59 (*p* < 0.001, Cohen's d = –0.67). Additionally, 42% (95% CI: 30–57%) expressed rather high or very high agreement with integrating LLMs into future treatment, while 18% (95% CI: 11–28%) expressed rather low or very low agreement. Together, the data show that patients generally have higher confidence in physicians over AI for most clinical capabilities, although AI is preferred for consultation time allocation; also, there is substantial interest in integrating LLMs into future treatment.

### 3.4 Confidence in AI is predicted by confidence in online information sources and not by age

Assuming that patients’ confidence in AI technologies might be influenced by age, we conducted a multiple linear regression analysis adjusted for age to identify factors associated with confidence in AI. The analysis revealed that only confidence in online information sources had a statistically significant association with confidence in AI (β = 0.72; 95% CI = 0.12–1.3; *p* = 0.018). The overall regression model was statistically significant, indicating a stable model (*p* = 0.049). Detailed results are presented in Table 2. Together, our findings show that patients' confidence in AI is strongly influenced by their trust in online sources and not by their age.

**Table 2:** Multiple linear regression model analyzing confidence in AI^a^

Coefficient	Beta	95% CI	*p* value
Age (yr)	0.01	–0.02, 0.05	0.4
Use of Google for medical Information?			
Yes	–	–	
No	0.32	–1.2, 1.9	0.7
Confidence in online sources?	0.72	0.12, 1.3	**0.018**
Affinity for artificial intelligence?	–0.09	–0.63, 0.46	0.8
Have you ever used ChatGPT or other LLMs for medQA?			
Yes	–	–	
No	1.7	–0.36, 3.8	0.10

AI = artificial intelligence; CI = confidence interval; LLM = large language model; medQA = medical question answering.

a Confidence in online sources was the single coefficient with a significant association with confidence in AI, *p* value (model): 0.04908.

## 4 Discussion

This study directly investigated the integration of AI, specifically LLMs, within a clinical setting offering patients hands-on experience, to assess the perception of patients on the clinical capabilities of AI. Our findings reveal that although patients appreciated the additional consultation time afforded by AI, human urologists were consistently preferred for tasks such as medical history taking, diagnostics, anxiety reduction, and treatment recommendations. Crucially, age did not affect confidence in AI, indicating that LLMs are accessible and user friendly across all age groups. These findings firmly support the role of AI as a powerful complement to—rather than a replacement for—human expertise in clinical care.

Our results solidify a general preference for AI as an assistive tool rather than a standalone decision-maker [18], [19], [26]. This underscores the irreplaceable value of the human elements—such as empathy, experience, and nuanced judgment. Our results suggest that patients may not want AI to take on responsibilities such as diagnosing, formulating treatment plans, or reducing their anxieties. However, LLMs hold promise as the next generation of medical information sources, aiding patients to navigate the often overwhelming and cluttered landscape of online medical information.

Enhanced patient information using simulations and multimedia tools has been proved to contribute to urological care by improving patient understanding, satisfaction, and engagement; supporting informed decision-making; reducing anxiety; and promoting treatment compliance [27]. LLMs, as natural language processors, can facilitate further enhancement of patient information by providing tailored, accessible explanations, facilitating more personalized and comprehensive patient education in urology. In general, AI is highly effective in processing and analyzing complex data. AI has, for example, shown benefits by improving early diagnosis, particularly in prostate and bladder cancer, through an advanced imaging analysis. Additionally, machine learning algorithms can predict patient outcomes and treatment success rates more accurately than conventional statistical methods, offering a range of applications in urological care [28].

Furthermore, LLMs can simplify complex medical information [27]. Research has shown that the complexity of medical information can be reduced significantly through the use of LLMs, particularly when prompted for improved readability [28]. LLMs could therefore be a key technology in streamlining patient communication and education, addressing current inadequacies in these areas.

The potential of LLMs to revolutionize medQA represents a promising advancement in how future medical knowledge is assessed in health care and how patient education is conducted [15], [16], [17]. Our study sought to understand how age might influence confidence in AI by adjusting the regression model accordingly. Contrary to our expectations, no significant age-related differences were found, indicating that LLMs could be effective across all age groups. Although only a small portion of patients, particularly younger ones, currently use LLMs for medical inquiries, the lack of age-related differences suggests broader applicability. Further research is needed to assess the user friendliness, especially among elderly populations.

Time constraints in patient communication are a pressing issue in health care systems. A study revealed that physicians in hospitals spend 4 min on average per patient on communication, underscoring the severe time pressure they face today [29]. Growing administrative demands, increased documentation tasks, and the need for multitasking are key contributors to this issue [29]. By 2050, the share of people over 65 yr in the European Union is projected to reach 30%, compared with 20% today, further exacerbating the need for more efficient health care solutions [30]. The demographic trend will widen the gap between the increasing demand for care and the time physicians can allocate. By offering unlimited interaction time, LLMs could help reduce the burden on health care providers. In practice, LLMs could serve as educational tools, enabling physicians to prioritize shared and informed decision-making while reducing the time spent on patient education.

Lastly, there is a noted gap between theoretical AI research and real-world evidence in health care [23]. Comprehensive and pragmatic trials, such as the IPaLLM study, are essential to thoroughly explore practical applications, user friendliness, effectiveness, and safety of LLMs, with a constant focus on patient-centered care.

This study has limitations from a potential social desirability bias [31], due to a direct questioning procedure. To address this, the presented findings are used in our ongoing study to design a collaborative follow-up study incorporating indirect questioning methods (eg, [26]). Additionally, the study may suffer from a sampling bias due to the predominantly male and elderly sample. However, this sample closely represents the desired typical patient population with urological diseases encountered in real-world settings. Furthermore, the controlled setting and standardized procedures of our study contribute to the validity and reliability of the study.

## 5 Conclusions

These findings firmly support the role of AI as a powerful complement to—rather than a replacement for—human expertise in clinical care. While patients favored human physicians for critical tasks, valuing the unique elements of human empathy and judgment, they appreciated the additional consultation time provided by AI. Age did not affect confidence in AI, indicating that LLMs could effectively be applied across age groups.

  ***Author contributions*:** Titus Josef Brinker had full access to all the data in the study and takes responsibility for the integrity of the data and the accuracy of the data analysis.

  *Study concept and design*: Carl.

*Acquisition of data*: Carl, Nguyen.

*Analysis and interpretation of data*: Carl, Winterstein, Wessels.

*Drafting of the manuscript*: Carl.

*Critical revision of the manuscript for important intellectual content*: Haggenmüller, Holland-Letz, Wessels, Brinker, Hartung, Gruene, Michel.

*Statistical analysis*: Carl, Holland-Letz.

*Obtaining funding*: None.

*Administrative, technical, or material support*: Wessels, Haggenmüller, Brinker.

*Supervision*: Wessels, Haggenmüller, Brinker.

*Other*: Creating graphic Illustrations: Winterstein, Hetz, Carl.

  ***Financial disclosures:*** Titus Josef Brinker certifies that all conflicts of interest, including specific financial interests and relationships and affiliations relevant to the subject matter or materials discussed in the manuscript (eg, employment/affiliation, grants or funding, consultancies, honoraria, stock ownership or options, expert testimony, royalties, or patents filed, received, or pending), are the following: Josef Brinker is the owner of Smart Health Heidelberg GmbH (Handschuhsheimer Landstr. 9/1, 69120 Heidelberg, Germany; https://smarthealth.de), outside the submitted work. Jakob Nikolas Kather reports consulting services for Owkin (France; producer of MSIntuit), Panakeia (UK), and DoMore Diagnostics (Norway), and has received honoraria for lectures by MSD, Eisai, and Fresenius, not related to this study. Frederik Wessels advises for AstraZeneca, Janssen, and Adon Health outside of the submitted work. The other authors have no conflicts of interest to declare.

  ***Funding/Support and role of the sponsor*:** The research is funded by the Ministry for Social Affairs, Health and Integration, Baden Württemberg, Germany. The funder had no role in study design, data collection and analysis, decision to publish, or preparation of the manuscript.
