# Supplementary material for: Comparing Patient’s Confidence in Clinical Capabilities in Urology: Large Language Models Versus Urologists
Source: Eur Urol Open Sci. 2024 Oct 23;70:91–8. doi: 10.1016/j.euros.2024.10.009 (PMC11538625; doi:10.1016/j.euros.2024.10.009)
Supplement: Supplementary Data 2 [file mmc2.docx]

Supplementary material for the manuscript: **Comparing Patient’s Confidence in Clinical Capabilities in Urology: Large Language Models vs. Urologists**

**B: pre-Interventional survey**

| **Questions** | **Answers** |
| --- | --- |
| Have you ever used the internet for medical questions? | - Yes - No |
| How often do you use the internet for medical questions? | - Daily - Weekly - Monthly |
| Please specify which online sources you have used. | *free text answer* |
| **How much do you agree with the following statement?** | |
| How high is your confidence in the internet to find accurate medical information? | - Very high - Somewhat high - Neutral - Somewhat low - Very low |
| How familiar are you with artificial intelligence? | - Very familiar - Somewhat familiar - Neutral - Somewhat unfamiliar - Not familiar at all |
| Have you heard of language models (for example ChatGPT, Gemini or other)? | - Yes - No |
| Have you ever used language models for medical questions? | - Yes - No |

**B: post-interventional survey**

| **Question** | **Antwort** |
| --- | --- |
| **Wie hoch ist Ihre Übereinstimmung mit der folgenden Aussage?** | |
| A physician is capable of capturing all relevant information from my medical history. | - Very high - Somewhat high - Neutral - Somewhat low - Very low |
| A language model is capable of capturing all relevant information from my medical history. | - Very high - Somewhat high - Neutral - Somewhat low - Very low |
| A doctor is likely to make the correct diagnosis. | - Very high - Somewhat high - Neutral - Somewhat low - Very low |
| A language model is likely to make the correct diagnosis. | - Very high - Somewhat high - Neutral - Somewhat low - Very low |
| A doctor is likely to recommend the correct treatment. | - Very high - Somewhat high - Neutral - Somewhat low - Very low |
| A language model is likely to recommend the correct treatment. | - Very high - Somewhat high - Neutral - Somewhat low - Very low |
| A doctor will recommend a treatment plan according to the current medical guidelines. | - Very high - Somewhat high - Neutral - Somewhat low - Very low |
| A language model will recommend a treatment plan according to the current medical guidelines. | - Very high - Somewhat high - Neutral - Somewhat low - Very low |
| A doctor can help me reduce my anxieties. | - Very high - Somewhat high - Neutral - Somewhat low - Very low |
| A language model can help me reduce my anxieties. | - Very high - Somewhat high - Neutral - Somewhat low - Very low |
| A doctor will give me all the relevant information for my treatment. | - Very high - Somewhat high - Neutral - Somewhat low - Very low |
| A language model will give me all the relevant information for my treatment. | - Very high - Somewhat high - Neutral - Somewhat low - Very low |
| A doctor will allocate enough time for me. | - Very high - Somewhat high - Neutral - Somewhat low - Very low |
| A language model will allocate enough time for me. | - Very high - Somewhat high - Neutral - Somewhat low - Very low |
| In the future, I would be happy to see language models as part of my treatment. | - Very high - Somewhat high - Neutral - Somewhat low - Very low |
